# Serum Lipoprotein (a) levels in acute coronary syndrome; Comparison of younger and elderly patients with healthy controls

**DOI:** 10.12669/pjms.35.6.377

**Published:** 2019

**Authors:** Sadaf Hanif, Bilqees Akhtar, Muhammad Naeem Afzal

**Affiliations:** 1 Dr. Sadaf Hanif, MD Cardiology. Department of Cardiology, King Edward Medical University, Lahore, Pakistan; 2 Dr. Bilqees Akhtar, FCPS. Department of Cardiology, King Edward Medical University, Lahore, Pakistan; 3 Dr. Muhammad Naeem Afzal, FCPS. Department of Medicine, King Edward Medical University, Lahore, Pakistan

**Keywords:** Acute coronary syndrome, Coronary vascular disease, Lipoprotein(a), Risk factor

## Abstract

**Objective:**

To compare and see the association of serum Lipoprotein (a) levels in younger and older patients suffering from acute coronary syndrome compared to healthy controls

**Methods:**

This case control study was conducted in department of cardiology, King Edward Medical University, Lahore from January to December 2015. Total 180 subjects (90 cases and 90 healthy controls, subdivided in 45 young and old in each group ≤/>45 years of age) were included in the study by non-probability purposive sampling. Patients presenting with acute coronary event and angiographically proven coronary vascular disease were considered cases while those with normal coronaries served as controls. Lp(a) was measured after ten hours fasting. Lp(a) >30 nmol/l) were considered as high. Data were entered and analyzed in SPSS 17. Independent sample t-test was used to compare the mean lipoprotein (a) in cases and controls.

**Results:**

The mean age of cases and controls was 48.02 ± 10.90 & 45.89±10.09 years respectively. Lipid profile was similar in both cases and controls except triglycerides that were higher in controls (p=0.024). The mean lipoprotein (a) in cases was 47.03 ± 45.47 and in controls was 29.69±23.10 (p-value 0.001). Mean Lp(a) level was significantly high in cases vs controls in young subjects, (50.15±55.62 vs 25.75±15.84, p= 0.006), while in old ones, difference was not statistically significant (43.92±32.69 vs 33.64±28.22, p= 0.114). The frequency of desirable, borderline high, high, and very high Lp(a) levels in cases was 23(25.6%), 12(13.3%), 27(30.0%) and 28(31.1%), while in controls, it was 26(28.9%), 31(34.4%), 17(18.9%) and 16(17.8%), (p-value 0.003). Chi-Square test showed significant association of high Lp(a) with coronary artery disease in younger cases vs controls (P=0.004) with OR 3.65 but not in older (p-value 0.358).

**Conclusion:**

Serum lipoprotein(a) is strongly associated with coronary vascular disease especially in patients younger than 45 years of age despite comparable LDL and HDL between cases and controls, making Lp(a) likely independent risk factor for coronary vascular disease.

## INTRODUCTION

Coronary vascular disease (CVD) is the major cause of morbidity and mortality and its prevalence is increasing in Pakistan.^1^ Among all conventional risk factors and lipid, one is lipoprotein(a), originally discovered by Berg in 1963, a variant of LDL whose amount and characteristics are genetically determined. It contains cholesterol-rich LDL particle along with apolipoprotein B100 and apolipoprotein(a). Lp(a) has high affinity to extracellular matrix in plaque; interferes with plasminogen activity due to its structural similarity with plasminogen and plasmin^2^, thus promotes atherogenesis in arterial vessel walls.

In past, there have been conflicting epidemiologic evidences about association of elevated Lp(a) with CVD. Helsinki Heart Study^3^ and the Physicians’ Health Study^4^, the two large epidemiological studies, did not favor an association. But later, Framingham Heart Study^5^, and ARIC (Atherosclerosis Risk in Communities) study^6^ proved positive association of Lp(a) with incident coronary artery disease over long term follow up. Kwon et al. established incremental association of Lp(a) with poor prognosis for major adverse coronary events in patient with coronary artery disease over three years follow up, suggesting it an important risk factor.^7^ Following cut-off levels of Lp(a) have been suggested to label risk stratification for coronary artery disease; optimal: ≤30mg/dl, high risk for ACS: 31-50 mg/dl, very high risk for ACS: >50 mg/dl. Cardiovascular risk is increased by 1.6 times if Lp(a) level is above 30 mg/dl.^8^ Case control studies from Pakistan have shown that patients with coronary artery disease have high Lp(a) levels as compared to age matched controls.^9^,^10^ In one study from Karachi, Lp(a) levels were high in children whose parents (especially fathers) had had MI, as compared to controls, strengthening a genetic association.^11^ But in these studies, controls were selected only on basis of negative history and normal ECG instead of normal coronary angiogram. Moreover, no study in Pakistan has looked into association of Lp(a) with CVD in different age groups. Therefore, we planned our study to evaluate Lp(a) as a cardiovascular risk factor in ≤/>45 years of age patients presenting with first episode of acute coronary syndrome and angiographically normal persons as controls, so as to consider Lp(a) in age stratified strategies to effectively target our high-risk patients with coronary vascular disease.

## METHODS

This case control study was conducted in department of Cardiology, King Edward Medical University/ Mayo Hospital, Lahore in January to December 2015, after approval by Institutional Review Board. (303/RC/KEMU/ Dec. 12^th^, 2012) Total of 180 subjects, 90 patients (45 cases of age ≤45 years (considered as young), 45 cases of age >45 years (considered as old) and 90 age-matched healthy controls (similarly divided in young and old) were enrolled by non-probability purposive sampling**.** Sample size of 90 in one group was calculated with 95% confidence interval and 5% margin of error taking 6% expected prevalence of coronary artery disease in Pakistani population. Patients with first episode of acute coronary syndrome who presented within the last seven days with suggestive history, ECG documented first acute (transmural or sub-endocardial) myocardial infarction, or unstable angina, and angiographically proven coronary artery disease were included in study. Subject having normal coronary arteries on angiogram (performed recently during work up for chest pain) served as control. Subjects with advance liver or kidney disease, or those taking drugs that may affect serum Lp(a) levels (like Niacin, Gemfibrozil, N-acetylcysteine, Estrogen, Tamoxifen, Omega-3 fatty acids, Prednisone and Neomycin) were excluded. Informed consent was taken both from patients and controls. The data was collected using a standard set of questions regarding demography, smoking, personal history of diabetes mellitus, hypertension and family history of ischemic heart disease. Height, weight and blood pressure was recorded as per standardized method. After ten hours of requested fasting overnight, three ml blood was collected from all study subjects from a large vein in antecubital fossa, in sitting position in morning hours. Blood samples were immediately centrifuged and serum isolated from them. QUANTIA Lp(a) assay on Abbott Architect platform ci8200 was employed for Lp(a) measurement that uses automated Latex enhanced technique for immunoassay. Following cut-offs of Lp(a) Levels were used to label risk stratification for coronary artery disease; optimal: ≤30mg/d, high risk for ACS: 31-50 mg/dl, very high risk for ACS: >50 mg/dl. Each participant was later treated according to his/her own clinical status. Report was conveyed to each patient. Healthy lifestyle was emphasized to everyone along with adherence to medication wherever indicated.

Data was entered in SPSS 17 version and analyzed. Normality of the data was determined by using *Kolmogorov–Smirnov test* (K–S test). Quantitative data was presented as mean ± SD. Qualitative variables were presented as frequency and compared on Chi-square test. For comparison of Lp(a) between cases and control group, independent sample t-test or Mann-Whitney U test was applied. A p-value <0.05 was taken as significant.

## RESULTS

In present study, total 180 patients were included. Cases were significantly obese than control. Lipid profile was statistically indifferent except mean triglyceride that was lower in cases (p-value 0.024) (Table-I). The mean LP(a) was significantly higher in cases as compared to controls (p-value 0.001) and it was significantly so in younger cases (p-value 0.006), but not in older ones (p-value 0.114). Mean Lp(a) was significantly high in non-diabetic subgroup (p=0.006) and those with no family history of ischemic heart disease (p=0.001), but not so in diabetics or with positive family history of IHD (Table-II).

**Table I:** Baseline characteristics of study subjects.

	Case	Control	p-value
Gender (M/F)	69/21	58/32	0.12
Height in cm	160.9+8.8	161.3+7.2	0.71
Weight in Kg	71.66+13.3	67.69+12.7	0.04
BMI	27.72+5.4	25.86+4.5	0.013
Blood pressure	119.9+12.7	120.3+12.7	0.83
Hypertension	39 (43.3%)	34 (37.8%)	0.45
Diabetes	23 (25.6%)	21 (23.3%)	0.73
Smoker	41 (45.6%)	36 (40%)	0.57
Family history of IHD	24 (26.7%)	23 (25.6%)	0.75
Total Cholesterol	189.87+29.96	190.06+33.58	0.96
HDL	37.82+5.28	37.73+4.68	0.90
LDL	108.18+18.46	113.11+27.19	0.16
Triglycerides	162.46+27.79	179.09+63.37	0.024

**Table II:** Comparison of mean Lp(a) level in total and different sub-groups.

	Cases (n=90)	Controls (n=90)	p-value
Total	47.03+45.47	29.69+23.50	0.001
≤ 45 years	50.15+55.62	25.75+15.84	0.006
> 45 years	43.92+32.69	33.64+28.22	0.114
Diabetic	47.54+42.18	32.36+17.68	0.119
Non-diabetic	46.86+46.84	28.77+24.74	0.006
Family H/O IHD	38.21+25.46	34.42+27.27	0.625
No family H/O IHD	50.24+50.61	28.07+21.47	0.001

Significantly higher percentage of cases (p-value 0.003) had undesirable (high, very high risk) levels of Lp(a) as compared to controls. In subgroups of young and old subjects, this association remained significant in young (p-value 0.004), but not in old group (p-value = 0.358). In total, those with high or very high Lp(a) levels had 2.7 times higher chances of having coronary artery disease as compared to those having desirable or borderline Lp(a) levels. Odds ratio and relative risk were also significant in younger subjects only (Table-III).

**Table III:** Association of categories of Lp(a) levels with presence of coronary artery disease.

	Cases ^a^	Controls ^a^	p-value	Odds Ratio ^b^	Relative risk ^b^
Age ≤45 years	17/14/14	31/9/5	0.004	3.65 (p=0.003) 95%CI: 1.5-8.7	2.00 (p-0.005) (95% CI: 1.2-3.2)
Age > 45 years	18/13/14	26/8/11	0.358	2.05 (p=0.09) 95%CI: 0.88-4.7	1.42 (p=0.09) (95%CI: 0.9-2.1)
Total	35/27/28	57/17/16	0.003	2.7 (p=0.001) 95%CI:1.5-4.9	1.67 (p=0.001) 95%CI: 1.2-2.3

a Values in each box in cases and controls columns represent actual number of subjects in category of optimal/high risk/very high risk Lp(a) levels respectively, (optimal ≤30 mg/dl, high risk: 31 - 50 mg/dl, very high risk: >50 mg/dl)

b Odds Ratio & relative risk were calculated taking high risk & very-high-risk categories as exposed.

Post-hoc Power calculation of the study shows that a sample of 184 was required to achieve 90% power of study. Our sample size of 180 persons (90 in each group) was sufficiently high to cross acceptable power of 80%.

## DISCUSSION

We studied association of Lp(a) with first acute coronary event in Pakistani patients with precise methodology. Our study has highlighted few important aspects. Firstly, it strengthens that Pakistani patients with CVD have significantly higher mean Lp(a) levels as compared to their counterpart with normal coronary arteries. Our study adds to the already existing similar evidence proven by Pakistani researchers^9^-^11^ but with improved methodology that both cases and controls were exactly classified on the basis of coronary angiography. The 30 mg/dl cutoff of high Lp(a) we used is consistent with recommendation generated by EPIC-Norfolk data that suggests that Lp(a) level between 24-36 mg/dl should be used to estimate risk of coronary artery disease.^12^ Recently, a large cross-sectional study in seven different ethnicities from INTERHEART project^13^ also established that patients with first acute MI have high mean Lp(a) levels. Persons with high Lp(a) levels (>50 mg/dl) had 48% higher odds of having acute MI as compared to controls, and this association was independent from other risk factors of coronary artery disease. South-Asians have high population attributable risk (9-10%) due to high prevalence of elevated Lp(a) in this population. The odds ratio of having an acute MI with Lp(a) >50 mg/dl was 1.48 in whole INTERHEART population, but it was not reported separately for South Asian population. Our study had higher odds of acute coronary syndrome (2.7 in whole study population, and 3.65 in patients younger than 45 years) with Lp(a) cutoff of 30 mg/dl as high. Taking this cutoff at 50 mg/dl also yielded OR 2.08 (not shown in results) that is also higher than collective INTERHEART data. This reflects important association of Lp(a) with acute coronary syndrome in Pakistani population, and more so in patients ≤45 years of age. A large scale cross sectional study PROMIS (Pakistan Risk Of Myocardial Infarction Study) involving 9015 Pakistani patients with acute MI and 8629 matched controls analyzed various biochemical and genetic variants with coronary artery disease, reported that OR of ischemic heart disease increases by 1.10 per 1 SD increase in Lp(a) concentration, even after adjusting for Lp(a) isoform and conventional lipids concentration.^14^

Our study adds that difference in mean Lp(a) concentration between cases of acute coronary syndrome and their controls is more marked and statistically significant in population younger than 45 years of age, as compared to those who are older than 45 years of age at first diagnosis of ischemic heart disease. A study from India also looked at the difference of Lp(a) in patients of acute MI in ≤45 years of age, and found high Lp(a) in cases as compared to control (33.84+23.69 vs 19.68+10.39 mg/dl, p<0.05) but selection of cases and controls was not based on angiography.^15^ Researchers from Australia investigated patients with premature coronary artery disease (<60 years) and found that Lp(a) >50 mg/dl was significantly associated with high SYNTAX and Gensini score, independent of other risk factors, depicting severe and complex coronary artery involvement across patient tertiles. Addition of raised LDL strengthened the association.^16^

In our study, the difference in Lp(a) levels in cases versus controls was statistically significant only in non-diabetics, while it was not-significant in Type-2 diabetics. This is in contrast with available literature. A study from Pakistan showed high levels of Lp(a) in Type-2 diabetics as compared to non-diabetics, though study subjects were not investigated for coronary artery disease.^17^ Results from BiomarCare consortium data, a large prospective population database of seven European cohorts involving 56804 participants with 24 years of follow up, highlight increased risk of coronary artery disease and major cardiac events with elevated Lp(a), in diabetics (HR 1.22 and 1.31 respectively) as compared to non-diabetics.^18^ Yet another study looking into the relationship of lipoproteins with beta cell function and cardiovascular parameters in diabetics discovered that Lp(a) has J-shaped curve association with cardiometabolic phenotype; low Lp(a) level is associated with reduced insulin sensitivity, poorer glycemic control, severe atherogenic dyslipidemia, microangiopathic disease but much lower coronary artery disease, while high Lp(a) level in diabetics is associated with macrovascular disease.^19^ In our study, although difference in Lp(a) concentration was quantitatively large between cases and controls in diabetic subgroup, (47.54±42.18 vs 32.36±17.68 mg/dl) but was statistically insignificant due to smaller number of subjects (23 each cases and controls); that needs a further study. Contrarily, statistically significant difference in Lp(a) in non-diabetics (p=0.006), reflect the situation in our society with larger number of participants (n=146: 67 each in cases and controls), emphasizing that Lp(a) may also be an important risk factor of coronary heart disease in non-diabetics in our population.

Our study also highlights the importance of Lp(a) in those with negative family history of ischemic heart disease. Majority (three forth) of our patients with CVD didn’t have any family history of coronary heart disease, yet high Lp(a) was strongly associated with coronary artery disease in these patients (50.24±50.61 vs 28.07±21.47 mg/dl, p-value: < 0.001). A recent study from USA has reported that Lp(a) may be an independent and important risk factor for asymptomatic coronary artery disease in African Americans with positive family history of ischemic heart disease.^20^ As Lp(a) levels are mostly genetically determined, it’ll be interesting to find out Lp(a) levels in parents and siblings of patients with coronary artery disease but negative family history. Our study, though neither designed nor powered to answer this question, however, suggests that while screening those with no family history of coronary artery disease, Lp(a) may be an important risk factor and must be used as screening tool in Pakistani population.

The most interesting fact in our study is that cases and controls had almost similar parameters of conventional lipid parameters but different Lp(a); this must have important impact in our clinical practice. Cases had even significantly lower triglycerides and marginally higher HDL but their coronaries were diseased; the culprit being high Lp(a). In other words, controls were rather protected from atherosclerotic effects of bad lipid profile because their Lp(a) level was low. This phenomenon was more prominent in younger ones. A hospital-based study from China involving 558 patients of acute MI and 1959 controls, both having normal LDL levels, revealed that Lp(a) was significantly different between those with diseased and those with normal coronaries. Taking a cut-off of 30 mg/dl, those with elevated Lp(a) had 1.52 times elevated chances of having acute MI.^21^ The FOURIER trial data showed that patients who are already on high intensity statins and LDL below 100 mg/dl, still had high chances of having major adverse cardiovascular events (22% in top quartile vs lowest quartile), based only on their high Lp(a) levels.^22^ Recent guidelines also emphasize checking of Lp(a) and other apo-B containing lipid particles if fasting lipid profile in an otherwise high-risk individual is not much beyond targets or patients are having recurrent events despite adequately on statins.^23^ It is thought provoking and raises important concerns regarding our population and practice. Will it not be appropriate that in addition to checking these factors, we also start doing Lp(a) routinely in our population especially the younger ones? This implies the need of prospective studies to discriminate high risk individuals on the basis of Lp(a). Secondly, we, being healthcare professionals, should refrain from reassuring patients only on the basis of their normal/near normal lipid profile, and this should be strictly implemented when we are risk-stratifying suspected patients of CVD presenting at age less than 45 years of age, because their lipid profile could be misleading when actually their Lp(a) levels are putting them in high risk category. Similarly, we should not ignore patients who are non-diabetic or don’t have family history of ischemic heart disease; they may be at high-risk for acute coronary events because of their high Lp(a) levels which must be screened. In other words, our study is a wake-up alarm for a necessary and urgent change in our screening patterns and need to build our own predictive scores to identify prospective patients of CVD at an earlier stage to target high risk population. We suggest that those subjects who are categorized at an equivocal risk of having coronary artery disease must have their Lp(a) checked in routine, and those with high Lp(a) levels should be given earlier appointment for decisive tests like CT angiography or invasive angiography irrespective of their history of diabetes or family history of IHD. Healthy lifestyle and high dose statins may attenuate the effects of high Lp(a) in these patients while some will still need PCSK9-inhibitors for their high Lp(a).^24^

There are certain strengths of our study. First is that sample size was adequate enough to reach 90% power of study. So, the results of our study are valid. Secondly, we used coronary angiography as necessary inclusion criteria for both cases and controls. Third and most important is that our study has provided a scientific evidence for age-based risk stratification of CVD on the basis of Lp(a) in addition to conventional risk factors. We however understand that case-control design does not establish causal risk association, so we suggest prospective studies to establish incremental prognostic value of Lp(a) in Pakistani patients at moderate and high risk of ischemic heart disease, especially younger ones, non-diabetics and with no family history of ischemic heart disease. A study from China has reported that among patients with non-obstructive coronary artery disease (detected by CT angiography) those who had high Lp(a) levels had high incidence of major adverse cardiac events in future.^25^ In our study, we cannot refute the presence of intra-luminal plaques in coronaries of those controls who have high Lp(a) levels. We suggest that those with high Lp(a) levels with normal coronaries should be followed up for incident cardiac events, and cases for recurrence of events. This will improve the predictive value of the model based on Lp(a) level. This may also help us identify those divergent persons who retain normal coronaries despite high Lp(a) levels, and may be of interest in studying genetic diversities at Lp(a) or its receptors.

## CONCLUSION

Serum Lp(a) is strongly associated with CVD. This association is present despite comparable LDL and HDL between cases and controls, making itself an important risk factor for CVD. The association of Lp(a) is stronger and statistically significant in patients younger than 45 years of age, in non-diabetics and with no family history of CVD. The findings imply necessary inclusion of Lp(a) in battery of investigations used for risk stratification of CVD in these patients.

### Authors’ Contribution:

**SH and BA** conceived, designed & editing of manuscript.

**SH** did data collection and manuscript writing.

**MNA** did statistical analysis and editing of manuscript, is responsible for integrity of research.

**BA** did review and final approval of manuscript.
